# Case Report: Association of a rare single nucleotide variant in the KCNH2 gene with drug-induced QT prolongation

**DOI:** 10.3389/fgene.2026.1715155

**Published:** 2026-02-24

**Authors:** Tianci Wang, Charlene R. Norgan Radler, Mohanakrishnan Sathyamoorthy

**Affiliations:** 1 Department of Medicine, Sathyamoorthy Laboratory, Burnett School of Medicine at TCU, Fort Worth, TX, United States; 2 Consultants in Cardiovascular Medicine and Science, Fort Worth, TX, United States; 3 Fort Worth Institute for Molecular Medicine and Genomics Research, Fort Worth, TX, United States

**Keywords:** arrhythmia, channelopathy, hERG, *KCNH2*, potassium ion channel, QT interval, quinolone, variant

## Abstract

**Background:**

Long QT Syndrome (LQTS) is characterized by prolonged QT intervals on electrocardiogram, which may progress into life-threatening polymorphic ventricular tachycardia and sudden cardiac death. Variants in the *KCNH2* gene have been associated with congenital LQTS, with thousands identified to date but very few clinically characterized.

**Objectives:**

To describe the rare single nucleotide variant *KCNH2* (NM_000238.4):c.1066C>T (p.Arg356Cys) associated with drug-induced QT prolongation and to assess its pathogenicity risk using *in silico* tools and protein structural modeling in accordance with American College of Medical Genetics and Genomics (ACMG) guidelines.

**Methods:**

Next-generation sequencing was performed for a patient presenting with drug-induced QT prolongation who was found to carry the rare *KCNH2* 1066C>T variant. Thirteen established gene discovery computational tools were employed to analyze the variant *in silico*. Additionally, structural modeling of the variant’s region within the wild-type protein was performed utilizing AlphaFold.

**Results:**

The clinical phenotype associated with the *KCNH2* 1066C>T variant has not been previously described in literature, except in combination with a variant in the *KCNQ1* gene. Computational analysis with a meta-predictor, REVEL, supported variant pathogenicity, while predictive modeling and AlphaMissense illustrated the uncertainty of structural impacts in a disordered region. Risk analysis of the variant performed utilizing ACMG guidelines and ClinGen criteria-specific recommendations resulted in an overall classification of “uncertain significance”.

**Conclusion:**

To our knowledge, this is the first study reporting a direct phenotype-to-genotype association between the *KCNH2* 1066C>T variant and drug-induced QT prolongation, supplemented by *in silico* analyses and ACMG-based variant risk stratification. Our study underscores the importance of recognizing genetic predisposition in drug-induced QT prolongation and motivate further investigation of *KCNH2* variants within the N-linker region.

## Introduction

Long QT syndrome (LQTS) is characterized by delayed cardiac repolarization seen as a prolonged QT interval on electrocardiogram (ECG). After heart rate correction, a prolonged QT interval (QTc) is considered as >460 ms in females, and >450 ms in males (Krahn et al., 2022). LQTS is dangerous due to its potential to progress into torsades de pointes, a life-threatening polymorphic ventricular tachycardia. LQTS is diagnosed by either an isolated QTc ≥500 ms, or Schwartz score ≥3.5 points, which incorporates the degree of QT prolongation, other ECG abnormalities, clinical history, and family history (Krahn et al., 2022; Schwartz and Ackerman, 2013). The most common presentation of LQTS is recurrent syncope, although patients may range from asymptomatic to experiencing dizziness, palpitations, dyspnea, or sudden cardiac death (Krahn et al., 2022).

LQTS may be congenital or acquired. Congenital LQTS consists of heritable diseases caused by abnormal cardiac ion channel function, affecting about 1 in 2,000-2,500 live births (Schwartz et al., 2009). Over 15 subtypes of congenital LQTS have been identified, based on the affected ion channel gene (Wilde et al., 2022). Subtypes 1–3 account for over 75% of cases and are caused by variants in *KCNQ1*, *KCNH2*, and *SCN5A*, respectively. Other, less commonly genes include *KCNE1*, *KCNE2*, *CACNA1C*, *CALM1-3*, and *CAV3* (Wilde et al., 2022). Congenital LQTS is known for its clinical heterogeneity and variable penetrance, complicating timely diagnosis and management (Krahn et al., 2022).

Acquired LQTS is more common and results from factors such as electrolyte disturbances, medications, or bradyarrhythmias (El-Sherif et al., 2018). The most frequent mechanism involves drug-induced blockade of the voltage-gated inwardly rectifying potassium channel encoded by *KCNH2* (El-Sherif et al., 2018). Despite preclinical screening for inhibition of this potassium current during drug development, many commonly used medications, such as antipsychotics, antibiotics, and antiarrhythmics, still carry QT-prolonging risk (Roden, 2016). Importantly, about a third of acquired LQTS patients have been found with pathogenic variants in LQTS-causing genes (Itoh et al., 2016). This suggests that medications may unmask an underlying genetic predisposition to LQTS, rather than being the sole cause of QT prolongation (Itoh et al., 2016).

The voltage-gated inwardly rectifying potassium channel KCNH2 conducts the rapid delayed rectifier potassium current (*I*_Kr_) during cardiac repolarization. It is a tetramer composed of a cytosolic N-terminus containing a PAS (Per-Arnt-Sim) domain, an N-linker, six transmembrane helices containing the voltage sensor, a selectivity filter, channel pore, and a cytosolic C-terminus containing a cyclic nucleotide-binding homology domain (CNBHD). The CNBHD does not bind any ligands but physically interacts with the PAS domain intracellularly to regulate channel gating (Foo et al., 2016). In native cardiac tissue, functional channels are hetero-tetramer of 1a and 1b subunits. Subunit 1a is the full-length channel protein, whereas 1b is an alternatively-spliced isoform replacing most cytosolic N-terminal domains with a unique 35-amino acid sequence. Co-assembly of both subunits is essential for channel function and cardiac repolarization (Foo et al., 2016).

*KCNH2* remains a major focus of LQTS research due to its implications in both congenital and acquired disease, with thousands of variants identified to date (Liu et al., 2025). In this study, we describe a patient without prior cardiac history who developed drug-induced LQTS and was found to carry a rare *KCNH2* variant of uncertain significance (VUS). *In silico* analyses and computational modeling of the region of interest were performed as preliminary assessment of the variant’s impact. A risk assessment was conducted based on American College of Medical Genetics and Genomics (ACMG) guidelines and ClinGen recommendations.

## Materials and methods

### Genetic testing

We utilized a commercially available panel targeting 42 genes associated with inherited arrythmias including LQTS, Brugada syndrome, catecholaminergic polymorphic ventricular tachycardia, arrhythmogenic right ventricular cardiomyopathy, along with other arrhythmias/channelopathies and sudden cardiac arrest (Supplementary Table S1) (Ambry Genetics, 2025). Details of the sequencing methods have been published previously and are provided in the Supplementary Material (Ambry Genetics, 2025).

### Variant analysis and interpretation

Information regarding the structure of voltage-gated inwardly rectifying potassium channel KCNH2 was obtained from UniProt (Nucleic Acids Research, 2025). Population frequency and ancestry-specific data were retrieved from Genome Aggregation Database (GnomAD), an international database aggregating data from a wide variety of large-scale data sequencing projects (Karczewski et al., 2020). All *KCNH2* N-linker variants were evaluated using public databases, including Human Gene Mutation Database (HGMD), Online Mendelian Inheritance in Man (OMIM), and ClinVar to assess phenotypic associations, pathogenicity evidence, and evolutionary conservation (National Library of Medicine, 2025; Stenson et al., 2014; Johns Hopkins University, 2025).

The Grantham score was used to predict the potential impact of the single amino acid substitution of the variant (Grantham, 1974). Additionally, variant risk interpretation was performed using American College of Medical Genetics and Genomics (ACMG) and the Association for Molecular Pathology (AMP) guidelines and Clinical Genome Resource (ClinGen) criteria-specific recommendations. Variant pathogenicity was classified using REVEL, and additional analyses performed using calibrated *in silico* tools (Tavtigian et al., 2008; Ioannidis et al., 2016; Tavtigian et al., 2006; Mathe et al., 2006; Ng and Henikoff, 2001; Choi et al., 2012; Choi and Chan, 2015; Shihab et al., 2013; Davydov et al., 2010; Cooper et al., 2005; Pollard et al., 2010; Sundaram et al., 2018; Katsonis and Lichtarge, 2014; Adzhubei et al., 2010; Kircher et al., 2014; Schubach et al., 2024; Feng, 2017; Carter et al., 2013; Pejaver et al., 2022; Pejaver et al., 2020), for which methodology has previously been described by our group (Norgan Radler et al., 2025). The selected tools along with their classification thresholds, mechanistic principles, clinical recognition, and limitations are provided in Supplementary Table S2.

The cryogenic electron microscopy (cryo-EM) assembly structure for the voltage-gated inwardly rectifying potassium channel KCNH2 (5VA2) was obtained from Research Collaboratory for Structural Bioinformatics Protein Data Bank (RCSB PDB) (Wang and MacKinnon, 2017; Berman et al., 2003). The monomeric wild-type predicted protein structure (Q12809) was obtained from AlphaFold Protein Structure Database (AFDB) and overlaid on a single monomer of the cryo-EM structure 5VA2 using the UCSF ChimeraX matchmaker tool (Varadi et al., 2022; Varadi et al., 2024). This method enables visualization of the arginine at position 356, which is absent from experimental structures in RCSB PDB.

The structure of *KCNH2* encoded protein containing the variant was predicted in AlphaFold3 using the NCBI FASTA reference sequence NM_000238.4, for which detailed methodology has been described by our group. Detailed data of AlphaFold modeling including confidence measures for the overall structure and local residues are detailed in Supplementary Table S3.

Informed consent was obtained from the individual participant involved in the study and is on file at CCMS-FW. Institutional Review Board approval was waived for this study.

## Results

### Clinical features

A 48-year-old female with a medical history of Crohn’s disease *status post* ileostomy was initially referred to our clinic for evaluation of a first degree atrial-ventricular block and premature ventricular contractions (PVC) noted on a pre-operative electrocardiogram (ECG) for ileostomy. She reported occasional palpitations but denied other cardiac symptoms or cardiac history including arrhythmias, coronary artery disease, or congestive heart failure. The patient was adopted with no available family medical history.

**FIGURE 1:**
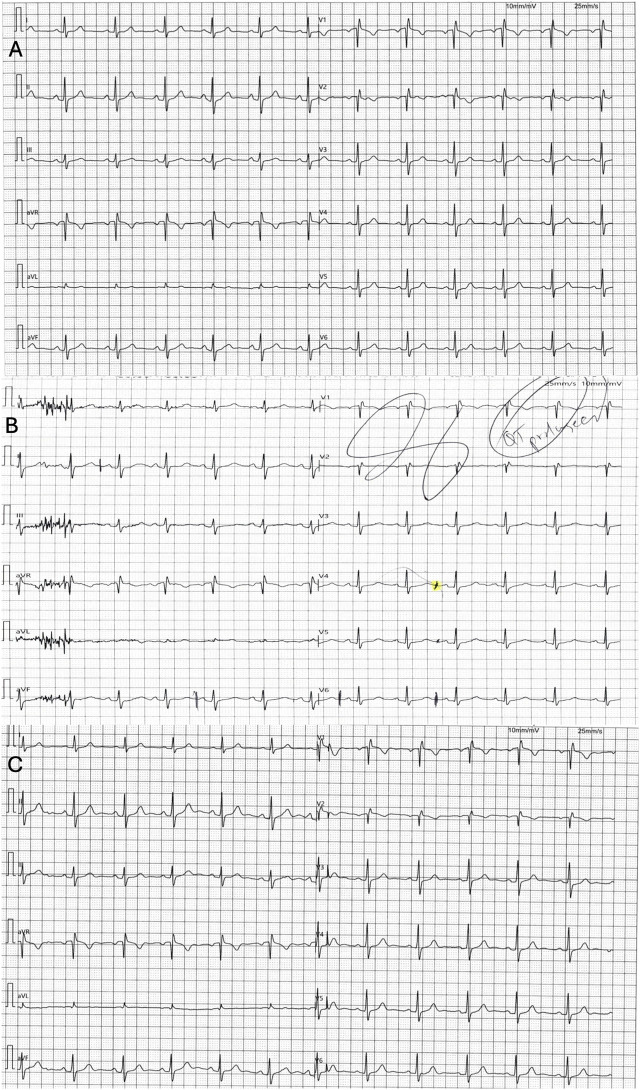
Patient ECGs at baseline (top) and during the long QT episode (middle) and after discontinuation of Ciprofloxacin (bottom). **(A)** Initial/baseline ECG obtained 3 months before the episode demonstrated sinus rhythm with early precordial qR transitions with a slightly elevated ST segment with a normal corrected QT interval of 435 ms. **(B)** 12-lead resting baseline ECG for stress test (note lead representation configured for stress ECG) demonstrated prolonged QT interval (QTc 545 ms). The patient had started ciprofloxacin 500 mg three times daily 5 days prior to this tracing. **(C)** ECG obtained 5 days after discontinuation of ciprofloxacin, demonstrating normal sinus rhythm with normalization of the QT interval to baseline (QTc of 428 ms).

Initial in-office ECG showed right bundle branch block (RBBB) with a qR pattern in lead V1, normal axis, and a normal QTc (Figure 1, top panel). Transthoracic echocardiography demonstrated a normal left ventricular ejection fraction (>70%) without evidence of right ventricular hypertrophy or indirect features of pulmonary arterial hypertension. A 48-h Holter monitor revealed a premature ventricular contraction and premature atrial contraction (PAC) burden of less than 1%, without any sustained atrial or ventricular arrhythmias.

The patient returned a few months later for further evaluation of a chest pounding sensation with dizziness and palpitations. She underwent a stress echocardiogram for age-appropriate ischemia risk stratification. Her stress ECG was negative for ischemia, but the corrected QT interval was noted to be significantly prolonged to 545 ms by Bazett formula (Figure 1, middle panel), which was not evident on her baseline ECG (Figure 1, top panel). We learned that she had recently initiated ciprofloxacin (5 days prior) and metronidazole for treatment of small intestine bacterial overgrowth. No further changes in medications were identified, and her Crohn’s disease was managed with as-needed hyoscyamine following her ileostomy. A comprehensive metabolic panel (CMP) ordered immediately showed no electrolyte abnormalities, with a normal potassium level of 4.1 mmol/L, and a calcium level of 9.4 mmol/L. The patient was known to have a normal magnesium level through review of historical labs. She was advised to immediately discontinue ciprofloxacin and educated on QT-prolonging agents. Magnesium oxide was initiated for stabilization of myocardial repolarization, and a mobile cardiac outpatient telemetry (MCOT) device was used for continuous ambulatory monitoring.

Following discontinuation of ciprofloxacin, ventricular arrhythmias or QT prolongation were not recorded during a 7-day MCOT monitoring period. In clinical follow-up at 1 week, her repeat ECG demonstrated normalization of the QT interval to her original baseline (Figure 1, bottom panel). Given the episode of a definitive drug-induced QT prolongation, we advised hypervigilant surveillance for any QT-prolonging medications and proceeded with clinical genetic testing to evaluate for any high risk pathogenic channelopathies. Biological family members were not available to provide additional clinical information or undergo family genetic studies.

### Genetic analysis

Genetic sequencing revealed a single variant of uncertain significance (VUS) in *KCNH2* (NM_000238.4):c.1066C>T (p.Arg356Cys). *KCNH2* has been definitively associated with long and short QT syndromes by Clinical Genome Resource (ClinGen) Gene Curation Expert Panels (GCEPs) (Clinical Domain Working Groups, 2025; Harrison et al., 2019; Richards et al., 2015; Clinicalgenome, 2025). A pathogenicity risk analysis was performed utilizing American College of Medical Genetics and Genomics (ACMG) guidelines along with ClinGen criteria-specific recommendations, with a resulting overall classification of “uncertain significance” (Table 1). The evidence utilized for each ACMG criterion is listed below.

**TABLE 1:** ACMG risk analysis incorporating ClinGen criteria-specific recommendations with supporting evidence.

Evidence type	Benign strong	Benign supporting	Pathogenic supporting	Pathogenic moderate	Pathogenic strong	Pathogenic very strong
Population Data	gnomAD FAF (0.004382%) does not support *BA1/BS1*. *BS2* is not applicable due to incomplete penetrance	​	​	gnomAD FAF (0.004382%) does not support *PM2*	Observed in an unrelated 8 y.o. female with LQTS (QTc 450 ms) and a *KCNQ1* VUS, whose reportedly unaffected father carried both variants and did not have CV diagnostic findings available. (Bora et al., 2023) Does not support *PS4*	​
Computational and Predictive Data (Pejaver et al., 2022)	​	REVEL score (0.659) does not support *BP4*. *BP1/BP3/BP7* are not applicable	REVEL score (0.659) is PP3 supporting +1 point	A different pathogenic variant does not exist at the same residue. Does not support *PM5*. *PM4* is not applicable	An established pathogenic variant with same amino acid change does not exist. Does not support *PS1*	*PVS1* is not applicable
Functional Data (cspec.genome, 2025; Brnich et al., 2019)	One RNA/protein metabolism assay showed no damaging effect. (O’Neill et al., 2024) Electrophysiology study has not been performed. Does not support *BS3*	​	Missense constraint Z-score is 2.5, thus benign missense variation is not significantly depleted. Does not support *PP2*	Not located in a mutational hotspot. Does not support *PM1*	Functional studies have not shown a damaging effect. Does not support *PS3*	​
Segregation Data	Biological family member data is unavailable. In one unrelated family, the proband’s reportedly unaffected father had a VUS in both *KCNH2* and *KCNQ1* and CV diagnostic findings were not available. (Bora et al., 2023) Does not support *BS4/PP1*					
*De novo* Data	​	​	​	Data is not available from the biological family and does not support *PM6/PS2*		​
Allelic Data	​	Not observed in *cis* or *trans* with another variant. Does not support *BP2*	​	*PM3* is not applicable due to AD inheritance	​	​
Other	​	An alternate molecular basis for disease was not found among 42 genes tested. Does not support *BP5*	Schwartz-score (Krahn et al., 2022; Priori et al., 2013) (0) does not support *PP4*: QTc 545 ms after exercise stress test in presence of QT-prolonging agent. No history of syncope or congenital deafness. Family history unknown	​	​	​
VARIANT CLASSIFICATION: UNCERTAIN SIGNIFICANCE (1 point)						

FAF, filtering allele frequency; CV, cardiovascular.

### Population data

The gnomAD GroupMax filtering allele frequency (FAF) for *KCNH2* (NM_000238.4):c.1066C>T is 0.004382% among South Asians, suggesting it is a very rare variant. The FAF is the maximum credible genetic ancestry group allele frequency (Harrison et al., 2019). Public databases were investigated for reports of the variant in unrelated patients with LQTS since case-control studies may not reach statistical significance for very rare variants. ClinVar contains five submissions for the variant, four of which are associated with LQTS or cardiac arrhythmias with unknown affected status (Supplementary Table S4) (National Library of Medicine, 2025). While the variant has also been reported in an unrelated eight-year-old female with LQTS (QTc 450 ms), she was found to have an additional *KCNQ1* VUS, resulting in uncertainty regarding each variant’s contribution. Although both variants were also carried by the patient’s reportedly unaffected father, we assessed this observation is not benign-supporting since LQTS has incomplete penetrance.

### Computational and predictive data

Computational data supports variant pathogenicity using a REVEL score of 0.659 per ClinGen recommendations for PP3 criteria (Supplementary Table S2). Additional *in silico* analyses were performed using well-established gene discovery tools to investigate conservation and predicted structural and functional impacts (Table 2) (Garcia et al., 2022).

**TABLE 2:** *In silico* analysis of the *KCNH2* (NM_000238.4):c.1066C>T variant, including score outputs and interpretations using thresholds established for gene discovery research.

Tool	Benign very strong	Benign strong	Benign moderate	Indeter-minate	Pathogenic supporting	Pathogenic moderate	Pathogenic strong	Pathogenic very strong
Supervised machine learning								
BayesDel	​		​	0.057	​	​	​	​
CADD v1.7	​	​	​	​	27.6	​	​	
REVEL	​	​	​	​	0.659	​	​	​
VEST4	​		​	0.601	​	​	​	
Structural/Physicochemical parameters								
Evolutionary Action	​		​	0.467	​	​	​	
MutPred2	​	​	0.341	​	​	​	​	​
PolyPhen2	​		​	​	0.998	​	​	
Sequence conservation								
PhyloP	​		​	3.357	​	​	​	
PrimateAI			​	0.534	​	​		
FATHMM			​	−2.33	​	​		
GERP++			​	​	​			
SIFT			​	0.04	​	​	​	

The GERP++ score for our variant (3.93) is not depicted, as it meets neither of the ClinGen validated thresholds for benign-supporting (−4.54 to 2.70) or benign-moderate (≤−4.54), A validated GERP++ classification category does not exist for this score.

Conservation analysis demonstrated that the arginine residue and its surrounding region are highly conserved (97%–100% identical) across nearly all examined mammalian species (Table 3). In non-mammalian species included in our analysis (birds and reptiles), this region showed significant divergence, although the arginine residue is consistently replaced by lysine, an amino acid with similar biochemical properties. Notably, the R residue is flanked by two acidic residues, glutamic acid and aspartic acid in mammals, and two glutamic acids in non-mammalian species, maintaining the local electrostatic environment. However, all four *in silico* tools that assess evolutionary sequence conservation (PrimateAI, PhyloP, SIFT, FATHMM) classified this variant as “indeterminant.”

**TABLE 3:** Sequence alignment diagram and amino acid conservation in selected vertebrate species from HGMD.

Organism	Protein ID	Alignment	Similarity
*Human*	NP_000229.1	346 D P F L A S P T S D **R** E I I A P K I K E R 366	100%
*Chimpanzee*	XP_024213853.1	388 D P F L A S P T S D **R** E I I A P K I K E R 408	100%
*Western gorilla*	XP_018886617.1	287 D P F L A S P T S D **R** E I I A P K I K E R 307	100%
*Sumatran orangutan*	XP_024106185.1	344 D P F L A S P T S D **R** E I I A P K I K E R 364	100%
*Rhesus monkey*	XP_028702300.1	346 D P F L A S P T S D **R** E I I A P K I K E R 366	100%
*House mouse*	NP_038597.2	348 D P F L A S P T S D **R** E I I A P K I K E R 368	100%
*Rabbit*	NP_001075853.1	331 D P F L A S P T S D **R** E I I A P K I K E R 351	100%
*Cow*	XP_024846603.1	359 D P F L A S P T S D **R** E I I A P K I K E R 379	100%
*Domestic cat*	XP_023106495.1	354 D P F L A S P T S D **R** E I I A P K I K E R 374	100%
*Big brown bat*	XP_027991322.1	354 D P F L A S P S S D **R** E I I A P K I K E R 374	97%
*Platypus*	XP_028933620.1	343 D P F L A S P T S D **R** E I I A P K I K E R 363	100%
*American alligator*	XP_019351032.1	259 D T F L A T P S G E **K** E I I A P T K V K D 279	44%
*Chicken*	XP_015136636.2	344 D A F L G A P S G E **K** E I I A P T K L K D 364	41%
*Peregrine falcon*	XP_027642489.1	286 D A F L A A P P G E **K** E I I A P T K L K D 306	42%

**FIGURE 2:**
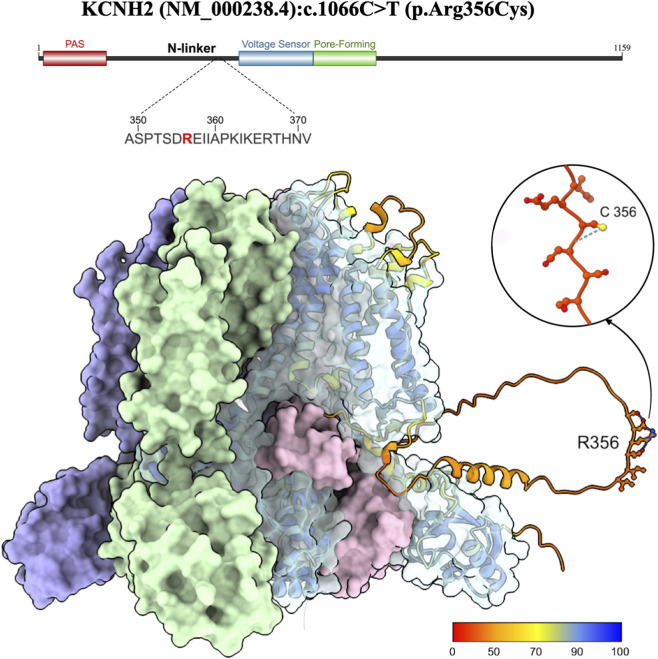
Structural visualization of the voltage-gated inwardly rectifying potassium channel KCNH2 from the RCSB PDB biological assembly 5VA2 (Wang and MacKinnon, 2017; Xie et al., 2022). The corresponding full-length predicted AFDB structure Q12809 overlays a single monomer, colored by per-atom predicted Local Distance Difference Test (pLDDT). Arginine at position 356 is labeled and visualized using ball stick style with heteroatom coloring. A color key located at the bottom right of the figure depicts pLDDT values, with higher values demonstrating higher confidence in the predicted structure.

The amino acid substitution of arginine to cysteine has a Grantham Score of 180 and is classified as a “radical change.” However, this variant was predicted as “likely benign” with a score of 0.312 by AlphaMissense, a deep-learning-based sequence analysis integrating structural context and population frequency. Other tools such as MutPred2 and PolyPhen-2 produced conflicting analyses of structural and functional consequences in the protein. Intriguingly, AlphaFold3 modeling revealed the variant is in a disordered region that is not well-characterized experimentally (Figure 2). The discordance between the amino acid substitutions and their predicted structural impact may partially result from the uncertain conformation of disordered regions. Detailed data of AlphaFold modeling are provided in Supplementary Table S3. Of note, the predicted structures include disordered regions in the N-linker, extracellular loops, and C-terminus which are usually excluded from experimental models and lower the global pLDDT.

### Functional data

The surrounding N-terminal linker region, which is not a known mutational hotspot, has 344 variants reported in ClinVar, most frequently in association with LQTS, followed by cardiovascular phenotype, cardiac arrythmia, and short QT syndrome (Supplementary Table S5). Most of the variants associated with LQTS have an unknown affected status and are classified as either VUS or as having conflicting classifications. Only three variants in this region are classified as likely pathogenic for LQTS; *KCNH2* 916G>T and 916G>C, which localize in a consensus splice site, and *KCNH2* 1015A>T. The variants *KCNH2* 916G>T and 916G>C have both been reported in patients with clinically confirmed LQTS in large-cohort sequencing studies, while *KCNH2* 1015A>T has been observed in a single patient with LQTS (Giudicessi et al., 2012; Tester et al., 2005). Human Gene Mutation Database (HGMD) reported 70 variants in the N-linker region, with similar associated phenotypes of LQTS and short QT syndrome, and additional conditions including sudden cardiac death, Brugada syndrome, sudden cardiac death, bicuspid aortic valve, sudden infant death, and catecholaminergic polymorphic ventricular tachycardia. Most variants were classified as disease-causing mutations (DM) for LQTS and Brugada syndrome (Supplementary Table S5) (Qiagendigitalinsights, 2025).

### Segregation/de novo/allelic data

LQTS is inherited in an autosomal dominant pattern with incomplete penetrance. The variant was not assessed for segregation with LQTS or *de novo* status because the patient’s biological family was unavailable. In one unrelated family, the proband’s reportedly unaffected father carried a VUS in both *KCNH2* and *KCNQ1* although cardiovascular diagnostic findings were not available (Bora et al., 2023). The variant was not observed in *cis* or *trans* with another variant.

### Other data

A Schwartz-score of zero was calculated based on: QTc 545 ms after exercise stress test in presence of QT-prolonging agent (Krahn et al., 2022; Priori et al., 2013). The patient did not have a history of syncope or congenital deafness, and has unknown biological family history. An alternate molecular basis for disease was not found among 42 genes tested.

## Discussion

In this study, we present novel clinical data on a rare single nucleotide variant, *KCNH2* (NM_000238.4):c.1066C>T, associated with drug-induced QT prolongation in a patient with no prior cardiac history, with *in silico* analyses and computational modeling to visualize the variant location and potential impact. With recent development of high-throughput automated expression and functional assessment assays for *KCNH2* variants, reporting of granular, variant-level clinical data remains valuable to assist in clinical risk stratification and validation of *in vitro* findings.

Loss-of-function variants in *KCNH2* may impair protein synthesis, trafficking, channel gating, or ion permeability, with over 80% resulting in trafficking defects (Delisle et al., 2004; Anderson et al., 2006; Anderson et al., 2014). Interestingly, variant impact appears to be influenced by domain location. Variants affecting the channel pore, particularly the S5-S6 helices, typically cause severe trafficking defects and often exert dominant-negative suppression of the WT protein, an effect less frequently observed in other domains. In contrast, N-terminal PAS domain and S4-S5 linker variants tend to alter channel gating, whereas C linker/CNBHD domain variants exhibit a mix of trafficking or gating defects depending on the specific locus affected (Foo et al., 2016; Anderson et al., 2006; Anderson et al., 2014). Clinically, pore-region variants are associated with a higher incidence of arrhythmic events than non-pore regions (Shimizu et al., 2009; Moss et al., 2002). A retrospective analysis of 2,826 *KCNH2* variants from publicly available gene databases showed that variants localized in the S5-S6 region including the selectivity filter, as well as the PAS domain, are associated with increased risk of cardiac events including torsades de pointes and sudden cardiac death (Liu et al., 2025).

This variant lies within the N-linker region which lacks a highly ordered structure and is generally considered more tolerant of amino acid changes (Anderson et al., 2014). The disordered structure also creates challenges in predicting local interactions and structural alterations via computational modeling. However, REVEL, a meta-predictor validated for clinical variant classification, provided evidence supporting pathogenicity based on ClinGen calibrated thresholds. Collectively, the combined clinical, population and computational evidence in this study supports a classification of “uncertain significance” for the variant.

To date, one prior report identified this variant in an eight-year-old female with a QTc of 450 ms who presented with dyspnea and palpitations (Bora et al., 2023). Notably, the patient also carried a VUS in *KCNQ1* (associated with LQT1) and both variants were also identified in the patient’s reportedly unaffected father (Bora et al., 2023). Additionally, a different variant affecting the same amino acid residue, *KCNH2* 1067G>A, was reported along with another variant, *KCNH2* 1682C>T, in a family with LQTS and sudden cardiac death, with both variants strongly segregating with the phenotype (Riuró et al., 2015). A recent high-throughput cell-surface expression study examined over 18,000 *KCNH2* variants including our variant of interest, and did not identify significant changes in cell surface expression compared with the wild-type (O’Neill et al., 2024). To our knowledge, variant-specific data is not available regarding effects on current conduction, gating kinetics, or interaction dynamics with wild-type 1a and 1b subunits, all of which are key parameters for understanding the variant’s functional consequences.

The N-terminal region, including the PAS domain and N-linker, has been implicated in regulating channel deactivation by physically interacting with the CNBHD domain, and facilitating the co-assembly of 1a and 1b subunits (Foo et al., 2016; Liu et al., 2016; Phartiyal et al., 2007). *In vitro* mechanistic studies on the N-linker region have been limited to segments that do not include the region harboring our variant, reducing their direct relevance to our work; nevertheless, several N-linker variants have been functionally characterized (Johnson et al., 2022). A 2009 study functionally examined eight variants identified in drug-induced LQTS patients, including an N-linker variant, *KCNH2* 1025A>T, found in a 70-year-old woman with markedly prolonged QTc (776 ms) and torsades de pointes following erythromycin exposure (Itoh et al., 2009). In Chinese Hamster Ovarian (CHO) cells, *KCNH2* 1025A>T did not have a detectable current when expressed alone and exerted a mild dominant-negative effect on the WT subunit when co-expressed (Itoh et al., 2009). Abnormal gating was also observed including a negative shift in the voltage dependence of inactivation and accelerated inactivation kinetics (Itoh et al., 2009). Similar patterns of mildly reduced current density and gating defects have been reported for other N-linker variants, partially consistent with a recent large-scale study suggesting variants in this region generally produce mild trafficking and conduction defects (O’Neill et al., 2024; Mattivi et al., 2020; Hayashi et al., 2020; Fodstad et al., 2006).

Heritable arrhythmogenic disorders affecting cardiac ion channels, such as LQTS and Brugada Syndrome, are generally transmitted in an autosomal dominant pattern with exceptions including Jervell and Lange-Nielsen syndrome. These cardiac channelopathies may demonstrate incomplete penetrance in which some individuals with a disease-causing variant do not manifest associated phenotypic features. Prior population-based estimates of LQTS penetrance are near 40%, with even highly penetrant founder mutations demonstrating phenotypic variability (Berge et al., 2008; Brink et al., 2005). Potential non-genetic modifiers contributing to these observed differences include sex and age, with males demonstrating greater risk of sudden cardiac death (Coll et al., 2017; Giudicessi and Ackerman, 2013). Age-related penetrance was previously demonstrated among children undergoing ajmaline provocation for Brugada Syndrome (McMillan et al., 2014; Conte et al., 2014). Additionally, exogenous factors such as excessive alcohol intake may unmask phenotypic electrocardiogram patterns in Brugada Syndrome. The incomplete penetrance and age-dependent expression observed in autosomal dominant cardiac channelopathies lend complexity to interpreting VUS findings in cases that may initially appear clinically silent.

In this case, the patient reported palpitations and dizziness which are a common presentation of LQTS, although many patients may remain asymptomatic (Millat et al., 2006). Given the risk of sudden cardiac death associated with LQTS and the widespread use of QT-prolonging medications, clinicians should maintain a high index of suspicion and pursue genetic screening in suspected cases or for first-degree relatives of affected cases (Al-Khatib et al., 2017). In accordance with the 2017 AHA/ACC/HRS guideline for the management of patients with ventricular arrythmias, individuals carrying the *KCNH2* 1066C>T variant should avoid QT-prolonging medications when possible; if required, careful monitoring of the QTc during treatment is needed with prompt discontinuation if marked QTc prolongation develops (Al-Khatib et al., 2017). Beta-blocker therapy is strongly recommended for patients with a resting QTc >470 ms, and is considered reasonable as long-term therapy in asymptomatic patients with resting QTc <470 ms (Al-Khatib et al., 2017). In patients with a significant symptom burden or symptoms refractory to beta-blocker treatment, more invasive therapy such as sympathectomy and implantable cardioverter defibrillator should be considered (Al-Khatib et al., 2017; Hauwanga et al., 2025).

Long-term clinical follow up and education on avoidance of QT-prolonging agents are important for all patients with LQTS, and individuals carrying variants of uncertain significance associated with LQTS, particularly when preliminary analyses such as *in silico* predictions suggest potential pathogenicity (Richards et al., 2015). The importance of continued surveillance is underscored by the population-level observation that the risk of sudden cardiac death increases steadily with age until reaching 60–80 years (Stecker et al., 2014; Stiles et al., 2020), likely reflecting the combined effects of underlying channelopathies and the increasing prevalence of cardiac comorbidities such as ischemic heart disease. Long-term clinical follow-up allows patients to receive updated information regarding potential variant reclassification (Muller et al., 2020), and also enables accurate and ongoing risk assessment as additional family and medical history emerge.

The QT prolongation in our patient was most likely triggered by ciprofloxacin, given its known QT-prolonging risks, and the observed strong temporal association and prompt resolution following its discontinuation. The patient’s other medications included metronidazole and hyoscyamine. While metronidazole carries a conditional QT-prolonging risk in the setting of excessive dosing, electrolyte abnormalities, or drug-drug interactions, these factors were not identified in this case (Wu et al., 2025; Crediblemeds, 2025). The potential contribution of several additional factors cannot be fully excluded. Notably, a serum magnesium level was not obtained at the time of her presentation. Isolated hypomagnesemia is a recognized cause of QT-prolongation in both healthy adults and susceptible populations (Moulin et al., 2015; Kieboom et al., 2016). Furthermore, mental stress, sympathetic activation, and autonomic conflict have also been shown to induce transient QT-prolongation, T wave abnormalities and arrythmias, particularly in genetically susceptible patients. Such factors could not be assessed in this case (Noda et al., 2002; Winter et al., 2018; Andrássy et al., 2007).

We acknowledge several limitations. Firstly, family pedigree and co-segregation analyses could not be performed due to the patient’s adopted status. Secondly, the reported genotype-to-phenotype association is based on a single patient and is limited by the potential influence of factors described above as well as other unidentified genetic modifiers. Thirdly, although *in silico* analyses offer valuable insight, critical information from electrophysiological data is not available. Future studies should explore *in vitro* expression and functional assessment of the variant via cell-surface ELISA, Western blotting and whole-cell patch-clamp to evaluate its impact on channel synthesis, trafficking, and function, to help clarify the mechanistic link between the genetic variant and the observed clinical phenotype.

## Conclusion

To our knowledge, this is the first study with detailed clinical data linking the rare variant *KCNH2* 1066C>T to drug-induced QT prolongation. Initial risk assessment of this variant utilizing population data and *in silico* analyses resulted in an overall classification of “uncertain significance” based on ACMG and ClinGen guidelines. Further functional characterization studies are warranted to further validate the pathogenicity of this variant and elucidate the mechanism of its phenotypes observed in this patient.

## Data Availability

The original contributions presented in the study are included in the article/supplementary material, further inquiries can be directed to the corresponding author. Deposition of genetic data into mandatory data repositories is limited by privacy restrictions for the single participant in this report.
