# Supplementary material for: Case Report: Association of a rare single nucleotide variant in the KCNH2 gene with drug-induced QT prolongation
Source: Front Genet. 2026 Feb 24;17:1715155. doi: 10.3389/fgene.2026.1715155 (PMC12971980; doi:10.3389/fgene.2026.1715155)
Supplement: Supplementary file 1 [file Table1.docx]

**Supplemental Methods**

*Genetic Testing*

Genomic deoxyribonucleic acid (gDNA) was isolated from the patient’s specimen using a standardized kit and quantified. Sequence enrichment of the targeted coding exons and adjacent intronic nucleotides was carried out by a bait-capture methodology using long biotinylated oligonucleotide probes and was followed by polymerase chain reaction (PCR) and Next-Generation sequencing.^1^ Additional Sanger sequencing was performed for any regions missing or with insufficient read depth coverage for reliable heterozygous variant detection.^1^ Variants in regions complicated by pseudogene interference, variant calls not satisfying depth of coverage and variant allele frequency quality thresholds, and potentially homozygous variants were verified by Sanger sequencing.^1^ Gross deletion/duplication analysis was performed for all genes using a custom pipeline based on read-depth from NGS data followed by a confirmatory orthogonal method, as needed.

*Variant Analysis and Interpretation*

AlphaFold structure predictions are freely available for both academic and commercial use under Creative Commons Attribution 4.0 (CC-BY 4.0) license terms.^2^ Molecular graphics and structure analyses were performed using UCSF ChimeraX version 1.10, developed by the Resource for Biocomputing, Visualization, and Informatics at the University of California, San Francisco, with support from National Institutes of Health R01-GM129325 and the Office of Cyber Infrastructure and Computational Biology, National Institute of Allergy and Infectious Diseases.^3^

Supporting files for molecular graphics and structure analyses performed on UCSF ChimeraX are provided below:

ChimeraX Session: 5VA2 with Q12809 Monomer Overlay

<https://doi.org/10.6084/m9.figshare.30735923>

ChimeraX Session: 5VA2 with R356C Monomer Overlay

<https://doi.org/10.6084/m9.figshare.30735935>

**Supplemental Tables**

| **Gene** | **Sequence** | **Gene** | **Sequence** | **Gene** | **Sequence** |
| --- | --- | --- | --- | --- | --- |
| *AKAP9* | NM_005751.4 | *HCN4* | NM_005477.2 | *RYR2* | NM_001035.2 |
| *ANK2* | NM_001148.4 | *JUP* | NM_002230.2 | *SCN1B* | NM_001037.4 |
| *CACNA1C* | NM_000719.6 | *KCND3* | NM_004980.4 | *SCN3B* | NM_018400.3 |
| *CACNA2D1* | NM_000722.2 | *KCNE1* | NM_000219.3 | *SCN4B* | NM_174934.3 |
| *CACNB2* | NM_201590.2 | *KCNE2* | NM_172201.1 | *SCN5A* | NM_198056.2 |
| *CALM1* | NM_006888.4 | *KCNE3* | NM_005472.4 | *SCN10A* | NM_006514.2 |
| *CALM2* | NM_001743.4 | *KCNH2* | NM_000238.3* | *SNTA1* | NM_003098.2 |
| *CALM3* | NM_005184.2 | *KCNJ2* | NM_000891.2 | *TBX5* | NM_000192.3 |
| *CASQ2* | NM_001232.3 | *KCNJ5* | NM_000890.3 | *TECRL* | NM_001010874.4 |
| *CAV3* | NM_033337.2 | *KCNJ8* | NM_004982.2 | *TMEM43* | NM_024334.2 |
| *DES* | NM_001927.3 | *KCNQ1* | NM_000218.2 | *TRDN* | NM_006073.2 |
| *DSC2* | NM_024422.3 | *LMNA* | NM_170707.2 | *TRPM4* | NM_017636.3 |
| *DSG2* | NM_001943.3 | *NKX2-5* | NM_004387.3 | *SCN3B* | NM_001035.2 |
| *DSP* | NM_004415.2 | *PKP2* | NM_004572.3 | *SCN4B* | NM_001037.4 |
| *GPD1L* | NM_015141.3 | *PLN* | NM_002667.3 |  |  |

**Supplemental Table 1:** The panel of 42 genes and associated NCBI sequences utilized for genetic sequencing of this patient.^1^ Of note, *KCNH2* variants in the manuscript are described based on the updated NCBI Ref Seq transcript NM_000238.4. The original genetic sequencing was performed using the prior transcript version NM_000238.3, which did not contain changes affecting the reported variant positions.

| **Tool** | **Pathogenicity Thresholds** | | **Clinical Recognition** | **Limitations** | **Core Principles** |
| --- | --- | --- | --- | --- | --- |
|  | **Developer Recommendation** | **ClinGen Calibration (PP3)** |  |  |  |
| Supervised Machine Learning (SML) | | | | | |
| **BayesDel** | MaxAF: > 0.069  No MaxAF:  > –0.057 | No MaxAF:  $\geq$ 0.13 | A | Potential over-fitting for known variants^4^ | Meta-predictor, may include or exclude maximum allele frequency |
| **CADD v1.7** | $\geq$ 20.0 | $\geq$ 25.3 | B | Does not integrate transcript or gene level annotations; supports GRCh37^5^ | Contrasts annotations of fixed/nearby fixed derived alleles in humans with simulated variants |
| **REVEL** | > 0.5 | $\geq$ 0.644 | A | Potential impact on accuracy or performance inflation by component predictors^6^ | Meta-predictor combining results from 13 tools |
| **VEST4** | > 0.5 | $\geq$ 0.764 | A | Like other SML tools, accuracy and bias impacted by training databases | Supervised machine-learning algorithm analyzing enrichment of functional mutations |
| Deep Learning | | | | | |
| **AlphaMissense** | $\geq$ 0.564 | N/A | C | Reduced performance for variants at residues predicted to be disordered^7^ | Deep learning tool using sequence and predicted structural context and population frequency data |
| Structural/Physicochemical Parameters | | | | | |
| **Evolutionary Action**^8^ | $\geq$ 0.5 | $\geq$ 0.685 | B | Availability of homologous sequences for evolutionary tree; residue interactions not integrated^8^ | Evolutionary importance of the mutated site and differences introduced by the amino acid change |
| **MutPred2** | > 0.5 | $\geq$ 0.737 | A | Predicted loss of protein-protein interactions may not be primary disease mechanism^9^ | Protein structure/function, evolutionary conservation |
| **PolyPhen2** | Probably damaging:  $\geq$ 0.909  Possibly damaging: 0.447–0.908 | $\geq$ 0.978 | B | Performance is sensitive to quality of homologous sequences used for conservation estimates^10^ | Protein structure/function, evolutionary conservation |
| Sequence Conservation | | | | | |
| **FATHMM** | $\leq$ –1.5 | $\leq$ –4.14 | B | Optional species-specific pathogenicity weights restricted to conserved protein domains^11^ | Evolutionary conservation |
| **GERP++** | > 2 | – | B | Thresholds may miss noncoding sites where mutations are under purifying selection^12^ | Genomic evolutionary rate profiling |
| **PhyloP** | > 2.27  (mammalian) | $\geq$ 7.367 | B | Power is sensitive to length of elements and sequence data availability; some contextual features are ignored; generalized assumptions about evolutionary selection^13^ | Nucleotide conservation scoring and identification of conserved elements |
| **PrimateAI** | >0.8 | $\geq$ 0.790 | B | Uses limited number of non-human primate genomes; assumes common variants in non-human primates are benign in humans^14^ | Deep neural network based on orthologous sequence alignment and conservation |
| **SIFT** | < 0.05 | $\leq$ 0.001 | B | High false positive rate at developer thresholds^15^ | Evolutionary conservation |

**Supplemental Table 2.** Pathogenicity thresholds, core principles, clinical recognition, and limitations for commonly used *in silico* tools. Thresholds are reported for gene discovery research and calibrated for ACMG clinical variant classification. Core principles and limitations are compiled from relevant literature and the UCSC Genome Browser.^16^ Clinical recognition (A/B) is provided by ClinGen 2022 recommendations for PP3/BP4 criteria: A) Recommended by ClinGen as a single tool valid for PP3/BP4 evidence at the calibrated threshold and relevant strength; and B) Valid to use for PP3/BP4 evidence at the calibrated threshold and relevant strength. Additionally, AlphaMissense showed C) 90% precision on the ClinVar dataset and similar performance to REVEL among a subset of cardiovascular genes. ^7,17^

| **Computational**  **Structure** | **Source** | **Reference Sequence** | **Global pLDDT** | **Local pLDDT AA# 356** | **H-bond**  **Length** |
| --- | --- | --- | --- | --- | --- |
| AF-Q12809-F1  (wild-type) | AFDB | NM_000238.4 | 63.81  (Low) | 33.09  (Very low) | D355-R356, 2.860 Å  R356-E357, 3.428Å |
| *KCNH2 1066C>T channel* | AF3 | NM_000238.4 | 55.85  (Low) | 31.50  (Very low) | D355-C356, 3.492Å  C356-E357, none |

**Supplemental Table 3.** Detailed data of AlphaFold modeling including pLDDT and length of H-bonds. AlphaFold structures are predicted as monomers and overlaid on a single chain of the experimental wild-type channel structure. The predicted structures contain both the N-linker and C-terminus which are disordered regions usually excluded from experimental models. Their inclusion in the predicted structures lowers global pLDDT. AFDB, AlphaFold Data Base; AF3, AlpaFold3 Server.

| **Condition** | **Classification** | **Number of Individuals*** | **Submission Year** |
| --- | --- | --- | --- |
| Cardiac arrhythmia | Uncertain significance | 1 | 2020 |
| Long QT syndrome | Uncertain significance | 1 | 2023 |
| Long QT syndrome | Uncertain significance | 5 | 2024 |
| Cardiovascular phenotype | Uncertain significance | 1 | 2025 |
| Not specified | Uncertain significance | 1 | 2025 |

**Supplemental Table 4.** Aggregate ClinVar submissions by condition for *KCNH2* 1066C>T . *Unknown affected status. The submitter reported that the participant’s phenotype information was not available at the time the variant was classified.

| **ClinVar** | | **HGMD** | |
| --- | --- | --- | --- |
| **Classification** | **Submissions # (%)** | **Classification** | **Submissions # (%)** |
| **Long QT Syndrome** | | | |
| Benign/Likely benign | 4 (1%) | DM | 47 (82.5%) |
| Uncertain significance | 312 (90%) |  |  |
| Likely pathogenic | 3 (1%) | DM? | 10 (17.5%) |
| Conflicting classifications | 26 (8%) |  |  |
| **Short QT Syndrome** | | | |
| Uncertain significance | 30 (86%) | DM | 1 (100%) |
| Conflicting classifications | 5 (14%) | DM? | 0 |
| **Brugada Syndrome** | | | |
| Uncertain significance | 1 (50%) | DM | 2 (100%) |
| Conflicting classifications | 1 (50%) | DM? | 0 |
| **Cardiac Arrhythmia** | | | |
| Uncertain significance | 39 (80%) |  |  |
| Conflicting classifications | 10 (20%) |  |  |
| **Cardiovascular Phenotype** | | | |
| Benign/Likely benign | 3 (3%) |  |  |
| Uncertain significance | 83 (84%) |  |  |
| Conflicting classifications | 13 (13%) |  |  |
| **KCNH2-related Disorder** | | | |
| Uncertain significance | 2 (100%) |  |  |
| **Hypertrophic Cardiomyopathy** | | | |
| Uncertain significance | 1 (50%) |  |  |
| Conflicting classifications | 1 (50%) |  |  |
| **Sudden Infant Death Syndrome** | | | |
|  |  | DM | 2 (40%) |
|  |  | DM? | 3 (60%) |
| **Sudden Cardiac Death** | | | |
|  |  | DM | 1 (50%) |
|  |  | DM? | 1 (50%) |

**Supplemental Table 5.** Summary of specified phenotypes with >1 submission in ClinVar (left, n=344 variants) and HGMD (right, n=70) for N-linker variants of *KCNH2*. For ClinVar: most submissions had affected status listed as “unknown”, and variants submitted with more than one phenotype are counted per submission. For HGMD, non-cardiovascular phenotypes and cardiovascular phenotypes with only one reported variant are not listed, including: catecholaminergic polymorphic ventricular tachycardia, bicuspid aortic valve, and autism. DM: disease-causing mutation; DM?: possible disease-causing mutation.

**References**

1. Genetic Testing for Long QT | Inherited Arrhythmia | LongQTNext & RhythmNext | Ambry Genetics. Accessed July 17, 2025. https://www.ambrygen.com/providers/genetic-testing/14/cardiology/longqtnext-rhythmnext

2. Varadi M, Bertoni D, Magana P, et al. AlphaFold Protein Structure Database in 2024: providing structure coverage for over 214 million protein sequences. *Nucleic Acids Res*. 2024;52(D1):D368-D375. doi:10.1093/nar/gkad1011

3. Meng EC, Goddard TD, Pettersen EF, et al. UCSF ChimeraX: Tools for structure building and analysis. *Protein Sci*. 2023;32(11):e4792. doi:10.1002/pro.4792

4. Feng BJ. PERCH: A Unified Framework for Disease Gene Prioritization. *Hum Mutat*. 2017;38(3):243-251. doi:10.1002/humu.23158

5. Schubach M, Maass T, Nazaretyan L, Röner S, Kircher M. CADD v1.7: using protein language models, regulatory CNNs and other nucleotide-level scores to improve genome-wide variant predictions. *Nucleic Acids Res*. 2024;52(D1):D1143-D1154. doi:10.1093/nar/gkad989

6. Ioannidis NM, Rothstein JH, Pejaver V, et al. REVEL: An Ensemble Method for Predicting the Pathogenicity of Rare Missense Variants. *Am J Hum Genet*. 2016;99(4):877-885. doi:10.1016/j.ajhg.2016.08.016

7. Cheng J, Novati G, Pan J, et al. Accurate proteome-wide missense variant effect prediction with AlphaMissense. *Science*. 2023;381(6664):eadg7492. doi:10.1126/science.adg7492

8. Katsonis P, Lichtarge O. Objective assessment of the evolutionary action equation for the fitness effect of missense mutations across CAGI-blinded contests. *Hum Mutat*. 2017;38(9):1072-1084. doi:10.1002/humu.23266

9. Pejaver V, Urresti J, Lugo-Martinez J, et al. Inferring the molecular and phenotypic impact of amino acid variants with MutPred2. *Nat Commun*. 2020;11(1):5918. doi:10.1038/s41467-020-19669-x

10. Adzhubei IA, Schmidt S, Peshkin L, et al. A method and server for predicting damaging missense mutations. *Nat Methods*. 2010;7(4):248-249. doi:10.1038/nmeth0410-248

11. Shihab HA, Gough J, Cooper DN, et al. Predicting the functional, molecular, and phenotypic consequences of amino acid substitutions using hidden Markov models. *Hum Mutat*. 2013;34(1):57-65. doi:10.1002/humu.22225

12. Huber CD, Kim BY, Lohmueller KE. Population genetic models of GERP scores suggest pervasive turnover of constrained sites across mammalian evolution. *PLOS Genet*. 2020;16(5):e1008827. doi:10.1371/journal.pgen.1008827

13. Pollard KS, Hubisz MJ, Rosenbloom KR, Siepel A. Detection of nonneutral substitution rates on mammalian phylogenies. *Genome Res*. 2010;20(1):110-121. doi:10.1101/gr.097857.109

14. Sundaram L, Gao H, Padigepati SR, et al. Predicting the clinical impact of human mutation with deep neural networks. *Nat Genet*. 2018;50(8):1161-1170. doi:10.1038/s41588-018-0167-z

15. Pejaver V, Byrne AB, Feng BJ, et al. Calibration of computational tools for missense variant pathogenicity classification and ClinGen recommendations for PP3/BP4 criteria. *Am J Hum Genet*. 2022;109(12):2163-2177. doi:10.1016/j.ajhg.2022.10.013

16. Cubuk C, Garrett A, Choi S, et al. Clinical likelihood ratios and balanced accuracy for 44 in silico tools against multiple large-scale functional assays of cancer susceptibility genes. *Genet Med*. 2021;23(11):2096-2104. doi:10.1038/s41436-021-01265-z

17. Ruiz M, Ochoa JP, Migoyo-Bettoni C, et al. Performance of AlphaMissense and Other In Silico Predictors to Determine Pathogenicity of Missense Variants in Sarcomeric Genes. *Circ Genomic Precis Med*. 2025;18(2):e004922. doi:10.1161/CIRCGEN.124.004922
